# Regulatory mechanisms of tetramethylpyrazine on central nervous system diseases: A review

**DOI:** 10.3389/fphar.2022.948600

**Published:** 2022-09-05

**Authors:** Yue Liu, Guang Yang, Wenqiang Cui, Yunling Zhang, Xiao Liang

**Affiliations:** ^1^ Xiyuan Hospital, China Academy of Chinese Medical Sciences, Beijing, China; ^2^ Guang’anmen Hospital, China Academy of Chinese Medical Sciences, Beijing, China; ^3^ Department of Neurology, Affiliated Hospital of Shandong University of Traditional Chinese Medicine, Jinan, China

**Keywords:** tetramethylpyrazine, central nervous system diseases, pharmacokinetics, protective mechanisms, ischemic stroke and reperfusion

## Abstract

Central nervous system (CNS) diseases can lead to motor, sensory, speech, cognitive dysfunction, and sometimes even death. These diseases are recognized to cause a substantial socio-economic impact on a global scale. Tetramethylpyrazine (TMP) is one of the main active ingredients extracted from the Chinese herbal medicine *Ligusticum striatum* DC*.* (Chuan Xiong). Many *in vivo* and *in vitro* studies have demonstrated that TMP has a certain role in the treatment of CNS diseases through inhibiting calcium ion overload and glutamate excitotoxicity, anti-oxidative/nitrification stress, mitigating inflammatory response, anti-apoptosis, protecting the integrity of the blood-brain barrier (BBB) and facilitating synaptic plasticity. In this review, we summarize the roles and mechanisms of action of TMP on ischemic cerebrovascular disease, spinal cord injury, Parkinson’s disease, Alzheimer’s disease, cognitive impairments, migraine, and depression. Our review will provide new insights into the clinical applications of TMP and the development of novel therapeutics.

## 1 Introduction

Central nervous system (CNS) disorders are a group of neurological disorders that affect the structure or function of the brain or spinal cord and are characterized by motor, sensory, speech, and cognitive impairment (Naval et al., 2011; Kundap et al., 2017). The increasing number of people affected by CNS diseases in recent years has undoubtedly caused a significant socio-economic burden on a global scale (Alavijeh et al., 2005). However, only a few efficient drugs are available to treat CNS diseases (Alavijeh et al., 2005; Kant, 2014). Several reasons contribute to this pitfall, such as the complex pathogenesis of the disease, the permeability of the blood-brain barrier (BBB), the side effects of CNS drugs, etc. Hence, there is a pressing need to develop novel therapeutic drugs effective against CNS diseases. Recent research has indicated that Chinese herbal medicines and their active ingredients can be promising in treating CNS diseases due to their beneficial role (Zhou et al., 2012; Liu et al., 2018; Fan et al., 2020).

**FIGURE 1:**
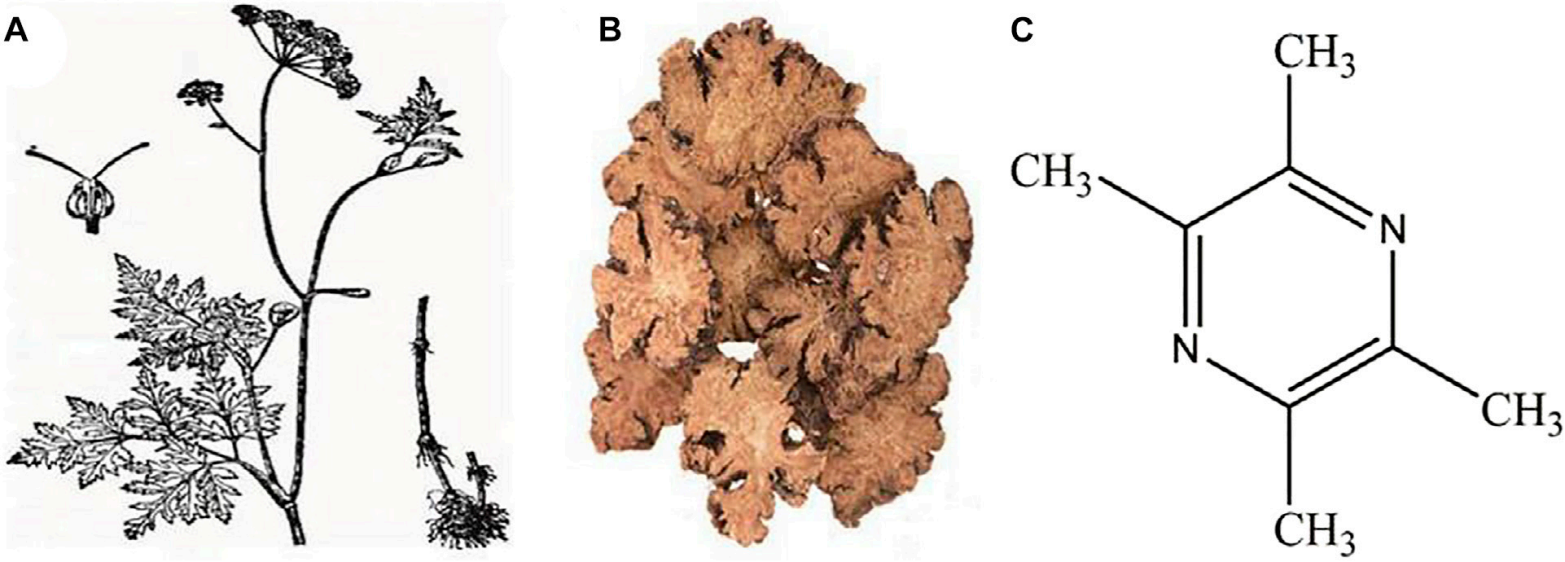
**(A)** *Ligusticum striatum* DC. (Chuan Xiong) plant; **(B)** Decoction pieces; **(C)** Chemical structure of tetramethylpyrazine [cited from Li J, Gong X. Front Pharmacol. 2022 Li and Tetramethylpyrazine (2022)].

Tetramethylpyrazine (ligustrazine, TMP) is an alkaloid monomer extracted from the dried rhizome of the Chinese herbal medicine *Ligusticum striatum* DC*.* (Chuan Xiong) [Figure 1 (Li and Tetramethylpyrazine, 2022)]. Chuan Xiong is a vital ingredient in Buyanghuanwu decoction, Houshiheisan, and Naoxintong capsules. All of them are classic prescriptions of traditional Chinese medicine (TCM) and have remarkable efficacy in treating cardiovascular and CNS diseases (Wang et al., 2014; Han et al., 2019; Gao et al., 2021). TMP is the more functional and structural representative of Chuan Xiong (Chang et al., 2007). Several studies have shown that TMP can exhibit multiple biopharmacological activities such as inhibition of platelet aggregation (Li et al., 2018), oxidative stress (Su et al., 2019), inflammation (Fan et al., 2011), apoptosis (Michel et al., 2016), and so forth. However, to date, the neuroprotective effects of TMP in CNS diseases have not been systematically reviewed. Our work tries to provide an overview of the pharmacological properties and therapeutic mechanisms of TMP associated with treating CNS diseases to facilitate its clinical application and provide a basis and direction for developing new drugs.

## 2 Pharmacokinetics of tetramethylpyrazine

Through various detection methods, the absorption, distribution, metabolism, and excretion of TMP *in vivo* have been extensively studied over the past few years. Studies showed that TMP was quickly absorbed in the human body with an absorption half-life of 0.15 h and the time required to reach its peak concentration in the blood was 0.51 h (Cai et al., 1989). Subsequently, TMP was rapidly and widely distributed *in vivo*, and the plasma protein binding rate of TMP was 64.64% (Ye et al., 2010). The blood pharmacokinetic parameters of unbound TMP conformed to the first-order rate and two-chamber open model (Tsai and Liang, 2001; Wang et al., 2012). In rats, different concentrations of TMP were detected in the kidneys, liver, fat, heart, spleen, muscle, and lungs (Wang et al., 2019). TMP penetrated the BBB and was distributed in various regions of the brain (cerebral cortex, brainstem, striatum, hippocampus, cerebellum, and midbrain) (Tsai and Liang, 2013), suggesting TMP may be effective in the treatment of cerebrovascular diseases (Liang et al., 1999).

In addition, the elimination of TMP was also quick. Following the intravenous injection, the pharmacokinetic profiles of TMP in rats were linear, and the half-life was about 35 min (Wang et al., 2019). It was also reported that the elimination half-life of TMP in rat blood and brain was 82.1 and 184.6 min, respectively (Tsai and Liang, 2013). 2-hydroxymethyl-3,5,6-trimethylpyrazine (HTMP) is the main active metabolite of TMP (Jiang, 1993). After administration of tetramethylpyrazine phosphate (TMPP) injection, high concentrations of HTMP were observed in the liver, lung, brain, kidney, heart, and spleen in rats, indicating that TMP is widely metabolized *in vivo* (Cao et al., 2013). Cytochrome P450 (CYP450) is the principal enzyme responsible for drug metabolism in the body and is distributed mainly in the liver microsomes (Schelstraete et al., 2018). About 15 enzymes belonging to the CYP1, CYP2, and CYP3 families are involved in the metabolism of foreign chemicals (Korobkova, 2015). A study further revealed that CYP3A4, 2C9, and 1A2 are the main CYP isoenzymes involved in the oxidative metabolism of TMP in human and rat liver microsomes (Tan et al., 2014). In summary, TMP has the pharmacokinetic characteristics of rapid absorption, wide distribution, and rapid elimination.

## 3 Drug delivery system of tetramethylpyrazine

The characteristic of short half-life makes TMP present a defect of low bioavailability, which limits its clinical application. Various methods have been used to enhance the TMP bioavailability, feasibility, and targeting. The drug delivery methods mainly include three crucial aspects.

### 3.1 Tetramethylpyrazine administration methods

A study showed that the bioavailability obtained by intranasal administration was significantly higher than that of intragastric administration (86.33% vs. 50.39%) (Meng et al., 2014a). TMP was absorbed more quickly through the nasal cavity, entered into the systemic circulation, and penetrated the BBB to target the brain (Jian et al., 2009; Meng et al., 2014b). Another clinical study compared the pharmacokinetics of transdermal patches and oral tablets of TMP in humans, indicating that the patch achieved the same therapeutic effect as oral administration (Shen et al., 2013). Also, it could prolong plasma TMP levels and lower drug fluctuations (Shen et al., 2013).

### 3.2 Combination of tetramethylpyrazine with other drugs

Borneol (BO), a crystalline isomer extracted from *Blumea balsamifera* (L.) DC. or *Cinnamomum camphora* (L.) J. Presl., effectively improves the bioavailability and blood concentration of co-administered drugs. It also increases the permeability of the BBB and promotes the drug to enter the brain, which is essential for its therapeutic role (Chen et al., 2019). BO can act as an effective adjuvant for delivering TMP to the brain. Studies have shown that the co-administration of BO could significantly increase oral absorption, areas under the concentration-time curve (AUC), and the concentration of TMP in the brain tissue (Yan-Yu et al., 2007; Liao et al., 2018; Wu et al., 2018).

### 3.3 Use of carrier systems for delivering drugs

Microemulsion (ME) is a new drug carrier system comprising a mixture of water, oil, and surfactants, and shows thermodynamic and kinetic stability (Karasulu, 2008). In mice, TMP-loaded microemulsion (TMP ME) presented a prolonged residence of time of the drug and markedly improved its overall targeting efficiency in the mice brain. A higher concentration of TMP was detected in mice’s brain, spleen, and lungs with the administration of TMP ME (Ma et al., 2013). In addition, ME-based transdermal delivery of TMP enhanced the TMP distribution rate to the brain and decreased its clearance rate from the brain (Zhao et al., 2011; Hu et al., 2019). The XBC microemulsion, a refined compound mixture of *Rhizoma ligustric Chuanxiong* extracts, BO, and TMP, enhances the bioavailability by increasing the half-life of TMP and prolonging the TMP residence time (Wang et al., 2012). The mean residence time of TMP in the brain also increased upon XBC microemulsion administration (Wang et al., 2012). Moreover, the lipid emulsion-based drug delivery system could significantly increase the mean residence time and half-life and decrease the clearance of TMP (Wei et al., 2012). Nanodrug delivery systems have received much attention recently because of their advantages, such as sustained, controlled release, and targeting (Altinoglu and Adali, 2019). According to another study, TMP-loaded nanoparticles modified with HIV-1 transcription factor (TAT) can boost the TMP half-life and membrane penetration rate, resulting in prolonged drug action and effective spinal cord targeting (Li et al., 2021a).

## 4 Effects of tetramethylpyrazine on central nervous system diseases

### 4.1 Ischemic cerebrovascular diseases

**FIGURE 2:**
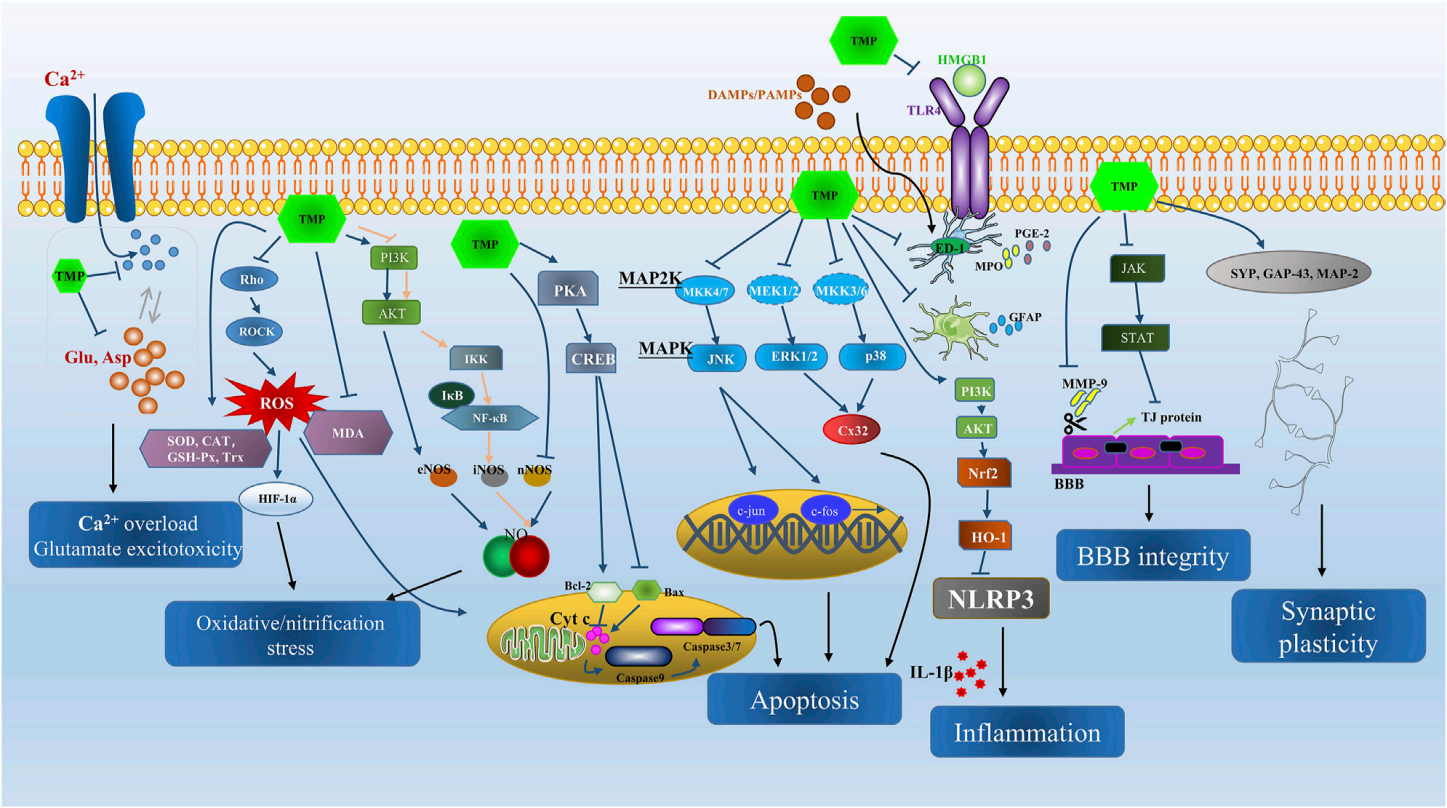
The summarization of neuroprotective effects and main signal pathways regulated by TMP in ICVD from both *in vivo* and *in vitro* studies.

Ischemic cerebrovascular diseases (ICVDs) are a major public health concern and affect human health worldwide. Among the ICVDs, ischemic stroke is the most common and one of the leading causes of high morbidity and mortality (Li et al., 2020a). Evidence suggests that thrombolytic therapy effectively treats ischemic stroke and decreases morbidity and mortality when timely provided (Man et al., 2020). Ischemia and reperfusion result in several cellular and biochemical consequences, including intracellular Ca^2+^ overload, excitatory amino acid poisoning, inflammatory response, oxidative damage, apoptosis, and so forth (Lo et al., 2003; Khatri et al., 2012; Radak et al., 2017; Lian et al., 2020; Lim et al., 2021). Novel treatments are urgently needed to prevent and treat the cerebral ischemia-reperfusion (I/R) injury. Prior reports indicate that TMP protects against the above-mentioned pathological mechanisms *in vivo* and *in vitro* (Figure 2).

#### 4.1.1 Inhibiting calcium ion overload and glutamate excitotoxicity

During ischemia and reperfusion, energy expenditure leads to the depletion of ATP and the imbalance of calcium homeostasis, causing several excitatory neurotransmitters (mainly glutamic acid) to be released into the synaptic space (Belov Kirdajova et al., 2020). Further, energy-dependent presynapses fail to reuptake the glutamate (Glu), resulting in its accumulation, a permanent influx of calcium ions, and neuronal death through excitotoxicity (Lipton, 1999; Hurtado et al., 2005; Chamorro et al., 2016; Mahmoud et al., 2019). As evident above, there is a synergistic effect between Ca^2+^ overload and the toxic effects of excessive excitatory amino acids.

TMP has been reported to suppress calcium mobilization from extracellular medium and intracellular stores (Liu and Sylvester, 1990). One study showed TMP as a calcium antagonist that blocked the influx of extracellular calcium through calcium channels and inhibited the release of intracellularly stored calcium in vascular smooth muscle cells (Pang et al., 1996). *In vitro*, using oxygen-glucose deprivation/reperfusion (OGD/R)-induced brain microvascular endothelium cells (BMECs) injury model, TMP treatment was found to significantly attenuate the Ca^2+^ overload in BMECs (Yu et al., 2019a). Also, in the permanent middle cerebral artery occlusion (MCAO)-induced cerebral ischemic model, TMP was shown to decrease the concentration of intercellular free [Ca^2+^]ions (Tang et al., 2012). The regulation of the level of excitatory amino acids (EAAs) during cerebral ischemia has always been the focus of research on the prevention and treatment drugs of cerebral ischemia. The concentrations of two EAAs, Glu and aspartic acid (Asp), were evaluated in MCAO/reperfusion (MCAO/R) rat models. The results indicated that ischemia increased the striatal concentrations of Glu and Asp, while TMP treatment notably abrogated the aberration (Han et al., 2014). Hence, it is apparent that the inhibition of Ca^2+^ overload and glutamate excitotoxicity is the key effect of TMP on cerebral ischemia and reperfusion, which could provide new insights into treating this disease.

#### 4.1.2 Anti-oxidative/nitrification stress damage

Free radicals include reactive oxygen species (ROS) and reactive nitrogen species (RNS), and oxidative/nitrification stress occurs when the production of ROS/RNS exceeds the clearance capacity of the antioxidant defense system (Diaz De Barboza et al., 2017). During ischemic stroke and reperfusion, ROS and RNS are generated in the brain tissue, activating mitochondria, death receptors, and endoplasmic reticulum stress pathways, ultimately mediating neuronal apoptosis (Stoll et al., 1998; Niizuma et al., 2009; Yuan et al., 2021). Thus, investigation of anti-oxidant/nitrification strategies becomes indispensable for treating cerebral ischemia and reperfusion.

In an OGD/R-induced BMECs injury model, TMP treatment relieved the oxidative stress by decreasing the concentrations of ROS and malondialdehyde (MDA) and upregulating the activities of antioxidant enzymes such as superoxide dismutase (SOD), catalase (CAT), and glutathione peroxidase (GSH-Px) (Yu et al., 2019a). Another study reported that TMP treatment enhanced the SOD levels and decreased the expression of lactate dehydrogenase (LDH), the earliest blood biochemical marker recognized during oxidative stress (Dave et al., 2016; Yang et al., 2017).

The transcriptional regulator hypoxia-inducible factor (HIF)-1α is a vital checkpoint of oxygen homeostasis, the expression of which rises rapidly after ischemia/hypoxia (He et al., 2021), and it plays a bidirectional role in ischemic stroke. On the one hand, it facilitates the adaptive response of cells in a hypoxic environment and modulates the expression of genes related to neurogenesis, angiogenesis, and cell proliferation (He et al., 2021). While on the other hand, it increases infarction volume and worsens nerve function by activating apoptosis and neuroinflammation, thus demonstrating a harmful effect (Chen et al., 2009; Cheng et al., 2014). Some researchers believe that low levels of HIF-1α can protect nerves, whereas the high expression of HIF-1α can cause cell death (Arumugam et al., 2018). Inhibiting the expression of HIF-1α in the early stages of ischemia is conducive to reducing cerebral edema and the number of apoptotic cells (Yeh et al., 2010). ROS plays a crucial role in promoting the stabilization of HIF-1α by inhibiting its ubiquitin degradation (Semenza, 2012; Zepeda et al., 2013). A study reported that ultrasound (US) exposure paired with TMP decreased intracellular ROS levels and inhibited the overexpression of HIF-1α in rat pheochromocytoma (PC12) cells. Thus, TMP exerted a neuroprotective effect by inhibiting the ROS/HIF-1α signaling pathway (Zhang et al., 2018).

Bone marrow-derived mesenchymal stem cells (BMSCs) transplantation has become a promising strategy for treating ischemic stroke (Li et al., 2007). However, transplanted BMSCs face the threat of oxidative stress in the ischemic microenvironment (Sun et al., 2012). TMP preconditioning could decrease intracellular ROS generation in hydrogen peroxide (H_2_O_2_)-induced BMSCs and is beneficial for cell survival in BMSCs transplantation therapy (Yan et al., 2017). Compelling evidence indicates that thioredoxin (Trx) plays an important role in the antioxidant defense mechanism (Rhee et al., 2001; Andoh et al., 2002; Kasuno et al., 2003). *In vivo* administration of TMP significantly decreased the volume of cerebral infarction and brain injury in MACO rats within 4 h post-reperfusion. This was likely achieved by upregulating the transcription of Trx (Jie et al., 2009).

Nitric oxide (NO), produced by inducible nitric oxide synthase (iNOS) and neuronal nitric oxide synthase (nNOS), is a typical representative of RNS. It can quickly react with superoxide (O_2_^−^) to produce a significant amount of peroxynitrite (ONOO-), resulting in nitrification stress (Pryor and Squadrito, 1995; Radi, 2004). On the contrary, NO produced by endothelial nitric oxide synthase (eNOS) exerts neuroprotective effects (Huang, 2004). Polymorphonuclear leukocytes (PMN) respiratory eruption induced by n-formylmethionyl-leucine-phenylalanine (fMLP) is often used to decipher the endogenous ROS and NO pathophysiology. Previous studies showed that TMP could scavenge the endogenous O_2_^−^ and reduce the generation of NO in a dose-dependent fashion in fMLP-stimulated PMN (Zhang et al., 2003). The results indicated that TMP could be used as a NOS activity regulator and ROS scavenger.

*In vitro*, TMP treatment could attenuate ROS overproduction and improve the expression level of eNOS (Yang et al., 2017). This effect is achieved through inhibition of the Rho/Rho-kinase (Rho-associated kinases, ROCK) signaling pathway, which plays a vital role in the pathogenesis of stroke (Koumura et al., 2011). Likewise, the increased levels of eNOS and NO with protective properties were noted after TMP treatment in OGD-induced human amniotic epithelial cells (HAECs) which may be related to the activation of phosphoinositide 3-kinase (PI3K) and serine/threonine kinase (Akt) signaling pathways (Ding et al., 2019). Furthermore, TMP therapy reduced infarct size in an MCAO-induced cerebral I/R rat model, with the underlying mechanism related primarily to reducing iNOS expression and preventing free radical generation (Sheu and Hsiao, 2006). Another study reported that TMP combined with astragaloside IV treatment modulated SOD activity upregulation and downregulated MDA content and iNOS activity (Yang et al., 2012). According to Zheng et al. (2013), TMP decreased the NO production and had toxic effects *in vitro* mediated by repressing the iNOS expression. The harmful effects were attributed to inhibition of the PI3K/Akt pathway and further suppressing IκB kinase (IKK) phosphorylation, IκB degradation, and nuclear factor κB (NF-κB) translocation, all of which are required for NO transcription (Zheng et al., 2013). It is worth noting that activation and inhibition of the PI3K/Akt pathway are beneficial to nitrification stress resistance (Zheng et al., 2013; Ding et al., 2019). The former targets to increase eNOS expression, while the latter targets to decrease the iNOS expression. However, in-depth research is needed to confirm the dual role of the PI3K/Akt pathway and the effects of TMP intervention.

It is well established that neurogenesis is necessary to repair the damaged brain (Rahman et al., 2021). Significant levels of NO are required to inhibit the proliferation of neural stem cells, and as nNOS decreases, cell proliferation and migration increase (Zhang et al., 2006; Zhang et al., 2007; Carreira et al., 2014). After TMP treatment in the MCAO-induced I/R rat model, Xiao et al. (2010) found that TMP could reduce infarction volume, neuronal loss, and water content by decreasing the expression of nNOS and further promoting cell proliferation and differentiation. These findings suggest that TMP can control antioxidant/nitrification stress damage during cerebral ischemia and reperfusion treatment. However, more detailed studies are required to confirm this theory.

#### 4.1.3 Mitigation of inflammatory response

Inflammation response involves in the pathogenesis of ischemic stroke and reperfusion. During ischemic stroke, oxygen deprivation and energy exhaustion lead to the accumulation of toxic metabolites in brain tissue, causing neuronal necrosis and apoptosis (Shah et al., 2019). Dead neurons release damage-associated molecular patterns (DAMPs) into the extracellular environment and produce large numbers of pro-inflammatory factors and chemokines, which rapidly activate microglia-mediated innate immune response (Xu et al., 2020). At the same time, reactive astrocytes promote the expression of pro-inflammatory factors such as interleukin- 6 (IL-6), tumor necrosis factor (TNF)-a, and IL-1β, and increase free radical ROS and NO levels (Liu and Chopp, 2016; Xu et al., 2020). Neutrophils are thought to be the primary source of free radicals during reperfusion (Funk et al., 2013; Jayaraj et al., 2019; Chen et al., 2021), and their counts are highest between 24 and 72 h after stroke. The activation of these immune cells can exacerbate the permeability of the BBB and destroy its integrity, aggravating nerve function damage (Huang et al., 2020).

Increasing evidence suggests that TMP exhibits anti-inflammatory effects against cerebral I/R. *In vivo*, TMP was proven to remarkably inhibit the immunoreactivity of ED-1, a microglia/macrophage marker protein (Liao et al., 2004; Kao et al., 2006). In the meantime, the activity of myeloperoxidase (MPO) and the production of prostaglandin E2 (PGE2) was significantly suppressed in the rat ischemic hemisphere, demonstrating the suppressive effect of TMP on the inflammatory cell activation and recruitment (Liao et al., 2004). Activation of astrocytes is characterized by increased glial fibrillary acidic protein (GFAP) in the blood (Dvorak et al., 2009). TMP combined with umbilical cord mesenchymal stem cells (ucMSCs) was found to rapidly downregulate the expression levels of GFAP and IL-1 (Cao et al., 2020).

The monocyte chemotactic protein-induced protein 1 (MCPIP1) is a suppressor of inflammation (Liang et al., 2010). Results from the cerebral I/R mice model induced by MCAO indicated that the TMP treatment effectively upregulates the expression of MCPIP1 and decreases the levels of inflammatory cytokines TNF-α, IL-1β, and IL-6 (Jin et al., 2021). Nuclear factor E2 correlation factor 2 (Nrf2) can regulate the oxidative stress and inflammation response by promoting the expression of the downstream heme oxygenase-1 (HO-1) gene (He et al., 2020). The PI3K/Akt pathway activation can promote Nrf2 nuclear translocation (Martin et al., 2004; Zhai et al., 2018). TMP sodium chloride injection was reported to enhance the expression of HO-1 by activating the PI3K/Akt/Nrf2 signal pathway (Zhu et al., 2022). At the same time, the expression levels of IL-1β, caspase-1, and NOD-like receptors family pyrin domain containing 3 (NLRP3) were significantly decreased by the treatment of TMP sodium chloride injection (Zhu et al., 2022).

High mobility group box 1 (HMGB1) is a highly conserved protein rapidly released extracellularly by the stimulation of pathogenic products (Andersson and Tracey, 2011). HMGB1 can bind to toll-like receptor-4 (TLR4), initiating the release of pro-inflammatory factors and causing tissue damage (Park et al., 2004; Fan et al., 2012). A clinical study showed that the serum HMGB1 levels in patients with cerebral ischemia were higher than in healthy volunteers (Goldstein et al., 2006). In the MCAO-induced cerebral ischemia rat model, TMP administration significantly inhibited neutrophil migration and suppressed the activities of HMGB1 and TLR4 (Chang et al., 2015). Interestingly, the inhibitory action of TMP on neutrophils was accompanied by increased expression levels of Nrf2 and HO-1 (Kao et al., 2013; Chang et al., 2015). Thus, according to the above findings, it is apparent that TMP could exert a strong anti-inflammatory response through the regulation of key targets or pathways in the pathogenesis of cerebral ischemia and reperfusion.

#### 4.1.4 Anti-apoptosis

Apoptosis is an essential form of programmed cell death (Wood and Bristow, 2010). A series of biochemical reactions after an ischemic stroke can cause apoptosis and necrosis. Mitochondria-mediated endogenous apoptosis plays a vital part in neurological impairment. Cytochrome c (Cyt c), a key regulator of apoptosis, activates the caspase enzyme during its release from the mitochondria to the cytoplasm, initiating the caspase cascade and apoptosis (Jemmerson et al., 2005; Yadav et al., 2020). B-cell lymphoma-2 (Bcl-2) and Bcl-2-associated X (Bax) are two crucial regulatory proteins of the mitochondria-mediated apoptotic pathway (Gross et al., 1999). A critical tumor suppressor gene, p53, regulates the expression of Bax to trigger apoptosis (Miyashita et al., 1995). TMP exerts anti-apoptosis effects mainly by inhibiting the pro-apoptotic protein Bax expression, promoting anti-apoptotic protein Bcl-2 expression, and blocking the caspase cascade.

In the cerebral ischemia-reperfusion rat model, administration of TMP combined with BO improved neuronal ultrastructure, reduced the numbers of apoptotic neurons by increasing the level of Bcl-2, decreased the expression of Bax, and inhibited the mRNA levels of p53 and caspase 3 (Yu et al., 2017). Yu et al. (2019a) also pointed out that TMP and BO work synergistically in regulating the expressions of Bcl-2, Bax, p53, and caspase 3 enzyme. In addition, *in vivo* and *in vitro*, TMP treatment suppressed DNA fragmentation, caspase-3 activation, and Cyt c release (Kao et al., 2006; Cheng et al., 2007). TMP can exert the above-mentioned regulatory effects in a variety of ways. The transient receptor potential ion channel subfamily C member 6 (TRPC6) is a Ca^2+^ permeable, non-selective ion channel widely distributed in neurons (Lin et al., 2013). Inhibiting the proteolysis of TRPC6 is favorable for combating neuronal death in cerebral ischemia (Johansson et al., 2015; Guo et al., 2017). TMP may protect neurons from OGD-induced apoptosis and inhibit the expression of caspase-3 by blocking the TRPC6 degradation (Shao et al., 2017). The cAMP-dependent protein kinase (PKA)/cAMP response element binding protein (CREB) signaling pathway plays a crucial regulatory role in apoptosis by promoting the Bcl-2 expression (Jiang et al., 2016). Cell experiments showed that TMP administration regulated the expression of Bcl-2 and Bax by activating the PKA/CREB signaling pathway (Tong et al., 2022). Further, pre-administration of TMP lowered the apoptosis rate of hippocampal neurons by inhibiting the JNK/MAPK signal pathway (Zhong et al., 2016). Connexins (Cx) are a family of membrane proteins that act as key players in cell death (Vinken et al., 2006). In OGD-induced hippocampal neurons, apoptosis and the expression of Cx32 were significantly increased (Gong et al., 2014). It was reported that TMP could reverse these changes by inhibiting the phosphorylation levels of extracellular signal-related kinases 1/2 (ERK1/2) and p38-MAPK (Gong et al., 2014).

#### 4.1.5 Protecting the blood-brain barrier

The BBB maintains a stable environment within the brain tissue. The tight junction (TJ) builds the structure and foundation of BBB. Cerebral ischemia and reperfusion increase BBB permeability, resulting in an enlarged infarct volume (Fan et al., 2018a). In the MCAO-induced cerebral ischemia/reperfusion rat model, TMP plays a neuroprotective role by reducing the infarct volume and BBB permeability, decreasing nerve score, and alleviating brain edema. The neuroprotective effect of TMP is related to attenuating the loss of TJ proteins, occludin and claudin-5 (Tan et al., 2015a). Further, the effects of TMP on enhancing the expression of TJ proteins are suggested through the inhibition of the activation of the JAK/STAT signaling pathway (Gong et al., 2019). Matrix metalloproteinases-9 (MMP-9) is an enzyme responsible for degrading the extracellular matrix and tight junctions (Candelario-Jalil et al., 2011). TMP treatment was also shown to preserve the BBB integrity by decreasing the levels of MMP-9 (Tan et al., 2015b; Jin et al., 2021).

#### 4.1.6 Enhancing synaptic plasticity

After ischemic stroke, the disruption of normal neuronal function leads to motor, memory, and cognition dysfunction. Evidence shows synaptic plasticity is involved in restoring neurological function after stroke (Yu et al., 2019b; Yepes, 2020). Synaptic morphology is the structural basis of synaptic function and plasticity (Harris and Weinberg, 2012). Synaptophysin (SYP) and growth-associated binding protein 43 (GAP-43) are considered molecular markers of synaptogenesis, and the increase in their expression levels is indicative of the synaptic number and function recovery (Hami et al., 2017; Merino et al., 2019). *In vivo*, TMP treatment improved the synaptic ultrastructure significantly, as observed by transmission electron microscopy. This was reflected in the modified main curvature of the synaptic interface, postsynaptic density thickness, and synaptic cleft width (Lin et al., 2021). Further, the expression levels of SYP and GAP-43 were upregulated upon TMP treatment (Lin et al., 2021). The microtubule-associated protein 2 (MAP-2) is a postsynaptic protein and a sensitive marker of ischemic stroke, the loss of which often indicates neuronal dysfunction (Li et al., 1998). In another *in vivo* study, TMP treatment markedly improved the expression levels of MAP-2 in peri-infarct area after stroke and alleviated the neurological deficits (Lin et al., 2015). The above studies show that TMP has a remarkable potential to enhance synaptic plasticity.

### 4.2 Spinal cord injury

Spinal cord injury (SCI), a central nervous system disorder defined by motor, sensory, or autonomic dysfunction, is caused by direct or indirect external factors that damage the spinal cord entirely or partially. SCI often predisposes the patients to limited mobility and various complications (Mcdonald and Sadowsky, 2002). Current treatments of SCI include early surgical decompression and fixation, mean arterial blood pressure augmentation, and corticosteroids (Karsy and Hawryluk, 2019). However, these strategies can only improve symptoms and reduce complications, and have a limited effect on nerve regeneration and functional restoration. Disorders of the spinal cord microenvironment after injury can lead to a series of pathophysiological changes (Fan et al., 2018b). Achieving rebalancing of the spinal cord microenvironment is a key strategy for repairing nerves (Fan et al., 2022). *In vivo* and *in vitro* studies have demonstrated that TMP has a regulatory effect on the spinal cord microenvironmen.

Macrophages and microglia are the vital effector cells of the inflammatory response that follows SCI (David and Kroner, 2011; Brockie et al., 2021). In acute spinal cord injury rat model, TMP has been found to decrease the expression of migration inhibitory factor (MIF), which may repair the injured spinal cord tissue (Xiao et al., 2012). The experiment designed by Hu suggested that TMP treatment could inhibit the expression of MIF, NF-кB, pro-inflammatory cytokine IL-18 and neutrophil infiltration and increase the level of NF-κB inhibitor and anti-inflammation cytokine IL-10 (Hu et al., 2013). Cyclooxygenase-2 (COX-2) and iNOS are downstream signaling molecules of the NF-κB signaling pathway, which induce the production of NO and PGE2, thus promoting cellular inflammatory response (Liou et al., 2014). *In vivo*, the TNF-α, IL-1β expression, and mRNA levels of COX-2 were upregulated in SCI-mice model. However, the TMP treatment could reverse the above mentioned changes, attenuating microglial activation and neutrophil infiltration (Shin et al., 2013).

The long-term neurological deficits after SCI are thought to be caused by extensive apoptosis of neurons and oligodendrocytes (Shi et al., 2021). *In vivo*, TMP visibly reduced the number of TUNEL-positive cells, downregulated the protein expression of cleaved caspase 3 and Bax, and upregulated the protein levels of B-cell CLL/lymphoma 2 like 2 (Bcl2l2) (Fan and Wu, 2017). Further, the microRNA-214-3p (miR-214-3p) expression levels decreased following the TMP treatment. The luciferase reporter gene assay and cell experiments showed that the miR-214-3p targets the 3′-UTR of the Bcl2l2 gene (Fan and Wu, 2017). Hence, it is reasonable to speculate that TMP promotes the expression of the anti-apoptotic gene Bcl2l2 by regulating the miR-214-3p levels, thereby alleviating neuronal cell apoptosis in SCI rats. TMP has also been found to reduce the expression of Fas ligand gene (FasL), phosphatase and tensin homolog (PTEN), and programmed cell death 4 (PDCD4), all of which are the contributors to apoptosis (Huang et al., 2016). The mechanisms may be related to enhancing the miR-21 expression levels (Huang et al., 2016). The above results showed that TMP is a promising candidate for SCI treatment.

### 4.3 Parkinson’s disease

Parkinson’s disease (PD) is a common CNS disease characterized by tremors and bradykinesia. Currently, the drugs for PD treatment mainly involve levodopa, dopamine agonists, monoamine oxidase B inhibitors, catechol-O-methyl transferase inhibitors, anticholinergics, and amantadine (Reich and Savitt, 2019). To further improve the clinical efficacy of the therapy and reduce the incidence of complications, combination strategies are intended for the prevention and treatment of PD.

**FIGURE 3:**
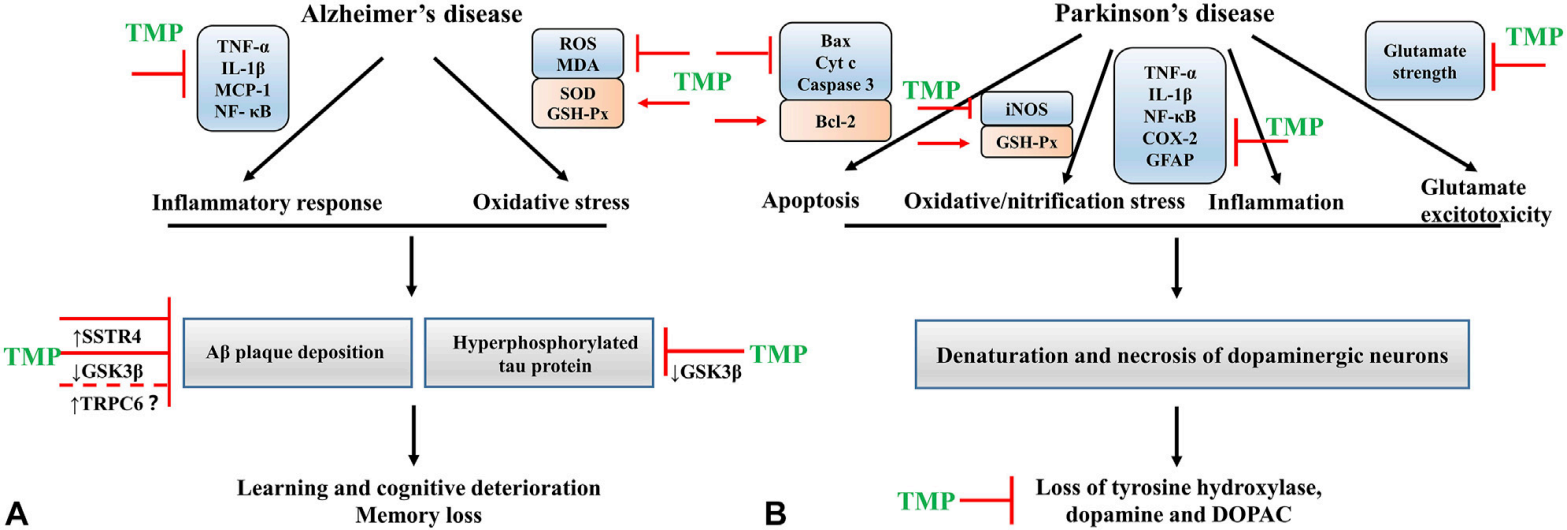
**(A)** The mechanisms of TMP intervention in AD; **(B)** The mechanisms of TMP intervention in PD.

The underlying pathological change in PD is the denaturation and necrosis of dopaminergic neurons in substantia nigra compacta (Xu et al., 2002), leading to a serious loss of tyrosine hydroxylase (TH) and dopamine (DA) (Kawahata and Fukunaga, 2020; Miller and O’callaghan, 2015). TMP has been proven to attenuate motor dysfunction, inhibit the decrease of TH expression, and prevent the reduction of DA and its metabolite- DOPAC (Lu et al., 2014). The regulatory effects of TMP in PD primarily involve inhibition of apoptosis, nitrification stress, inflammation, and glutamate excitotoxicity. Neurotoxin 1-methyl-4-phenyl-1,2,3,6-tetrahydropyridine (MPTP) causes PD-like behavioral symptoms. Thus, the MPTP model has been used for experimental studies of PD. In an MPTP-induced PD rat model, TMP prevented the decrease of GSH, downregulated Bax expression, upregulated Bcl-2 expression, and inhibited the release of Cyt c and the lysis of caspase 3 (Lu et al., 2014). In addition, TMP could suppress the upregulation of TNF-α and IL-1β and modulate the glutamate levels *in vivo* (Zhao et al., 2014)**.** In the rotenone-induced PD rat model, TMP attenuated the ratio of Bax/Bcl-2, inhibited the activation of caspase 3, and downregulated the expression levels of NF-кB, iNOS, COX-2, and GFAP (Michel et al., 2016). The findings reported in these studies demonstrate that TMP has a favorable effect on the treatment of PD (Figure 3).

### 4.4 Alzheimer’s disease

Alzheimer’s disease (AD) is a progressive neurodegenerative disease characterized by cognitive deterioration and memory loss, threatening human health and public health systems. Despite the serious public health problems the disease poses, only five drugs have been approved to treat AD (Briggs et al., 2016). Among them, donepezil, rivastigmine, galantamine and memantine are the most commonly used drugs that effectively relieve AD symptoms. Though they have side effects such as gastrointestinal, fatigue, dizziness and muscle cramps (Briggs et al., 2016). Given the rising prevalence of AD, there is an urgent need to develop new therapeutic drugs.

Evidence shows that *β* amyloid (Aβ) plaque deposition and hyperphosphorylated tau protein are the typical pathological changes observed in AD (Lane et al., 2018). *In vivo* studies, TMP has been proven to improve learning and cognitive function in AD models by lowering the Aβ deposition and tau phosphorylation levels (Huang et al., 2021; Zhang et al., 2021). The sequential cleavage of amyloid-beta precursor protein (APP) by *β* and γ-secretases is critical in Aβ production (Xiaojie and Weihong, 2013). One study demonstrated that TRPC6 could reduce the generation of Aβ by inhibiting the cleavage by γ-secretase (Wang et al., 2015). As mentioned before, TMP has a regulatory effect on TRPC6 (Shao et al., 2017). However, this effect is unknown in AD diseases and needs to be further addressed. The glycogen synthase kinase-3β (GSK-3β) is a multi-potential serine/threonine kinase that plays a crucial role in several cellular processes, including cell proliferation, differentiation, transformation, and apoptosis (Demuro et al., 2021). The activation of GSK3β contributes to memory loss, tau hyper-phosphorylation, increased Aβ deposition, and local plaque-associated microglial-mediated inflammatory responses (Hooper et al., 2008). In the AD rat model, TMP treatment inhibited the activity of GSK-3β and restored the function of cholinergic neurons. This neuroprotection provided by TMP may be mediated by the activation of Akt (Lu et al., 2017).

Neuroinflammation is a pathological feature of AD. Aβ can activate the microglial cells to release proinflammatory and cytotoxic factors, thus aggravating the inflammatory response of the CNS (Lue et al., 2010). In cultured microglial cells stimulated with Aβ_25-35_ and interferon (IFN)-γ, TMP could suppress the production of microglial proinflammatory mediators TNF-α, IL-1β and monocyte chemotactic protein 1 (MCP-1), and inhibit the activity of NF-κB (Kim et al., 2014). TMP pretreatment also decreased the ROS production in microglia (Kim et al., 2014). Somatostatin (SST) is a circular polypeptide that binds to somatostatin receptors (SSTR) on the cell membrane and is widely distributed in the CNS and peripheral tissues. It plays a vital role in memory, cognition, and emotional function (Arimura et al., 1975; Patel and Reichlin, 1978; Finley et al., 1981; Epelbaum et al., 2009). SST enhances the activity of neprilysin, an enzyme that degrades Aβ (Rofo et al., 2021). A study showed that the SST and SSTR protein levels in the cerebral cortex of Alzheimer’s patients were significantly lower than that in healthy individuals. Thus these proteins could serve as markers of the disease (Kumar, 2005). *In vivo,* TMP treatment improved the learning and memory function, attenuating AD mice’s cognitive impairment. The potential mechanism might be related to reducing the ubiquitination of SSTR4, thus increasing its levels in the system (Weng et al., 2021). Apart from these, researchers have designed the ligustrazine phosphate (LP) transdermal ethosomal system, and evaluated the effect of LP on AD in scopolamine-induced amnesia rats. The results showed that the LP transdermal ethosomal system could markedly increase SOD and GSH-Px activities, decrease MDA levels and reverse these activities/levels to the similar status of the normal rats (Shi et al., 2012) (Figure 3).

### 4.5 Cognitive impairment

Vascular dementia (VD) is the second leading type of dementia after Alzheimer’s disease. The clinical manifestations of VD include memory loss, emotional and behavioral changes, cognitive impairment, and executive dysfunction. Chronic cerebral ischemia, hypoxia, and hypoperfusion are the important causes of VD (Iadecola, 2013). Studies have demonstrated that TMP has a protective effect against VD, primarily through apoptosis inhibition and synaptic plasticity facilitation. In the bilateral common carotid artery occlusion (BCCAO) surgery-induced VD rats, TMP therapy modulated the pro-and anti-apoptotic indicators, inhibited the elevated MCP-1 and homocysteine (Hcy) levels, and suppressed the lowered levels of brain-derived neurotrophic factor (BDNF) (Zhao et al., 2017). TMP also significantly reduced the ratio of Bax/Bcl-2 protein and cleaved caspase-3 expression in OGD PC12 cells (Zhao et al., 2017). In the BCCAO stimulated rats, chronic restraint stress (CRS) is measured by the novel object recognition test, social interaction test, and Barnes maze paradigm. It was discovered in these rats that TMP treatment improved cognition, sociability, and learning/memory impairments, by increasing the expressions of synapse-related proteins PSD95, SYN, GAP43, and SYP by activating the TrkB/ERK/CREB signaling pathway (Tan et al., 2021).

**FIGURE 4:**
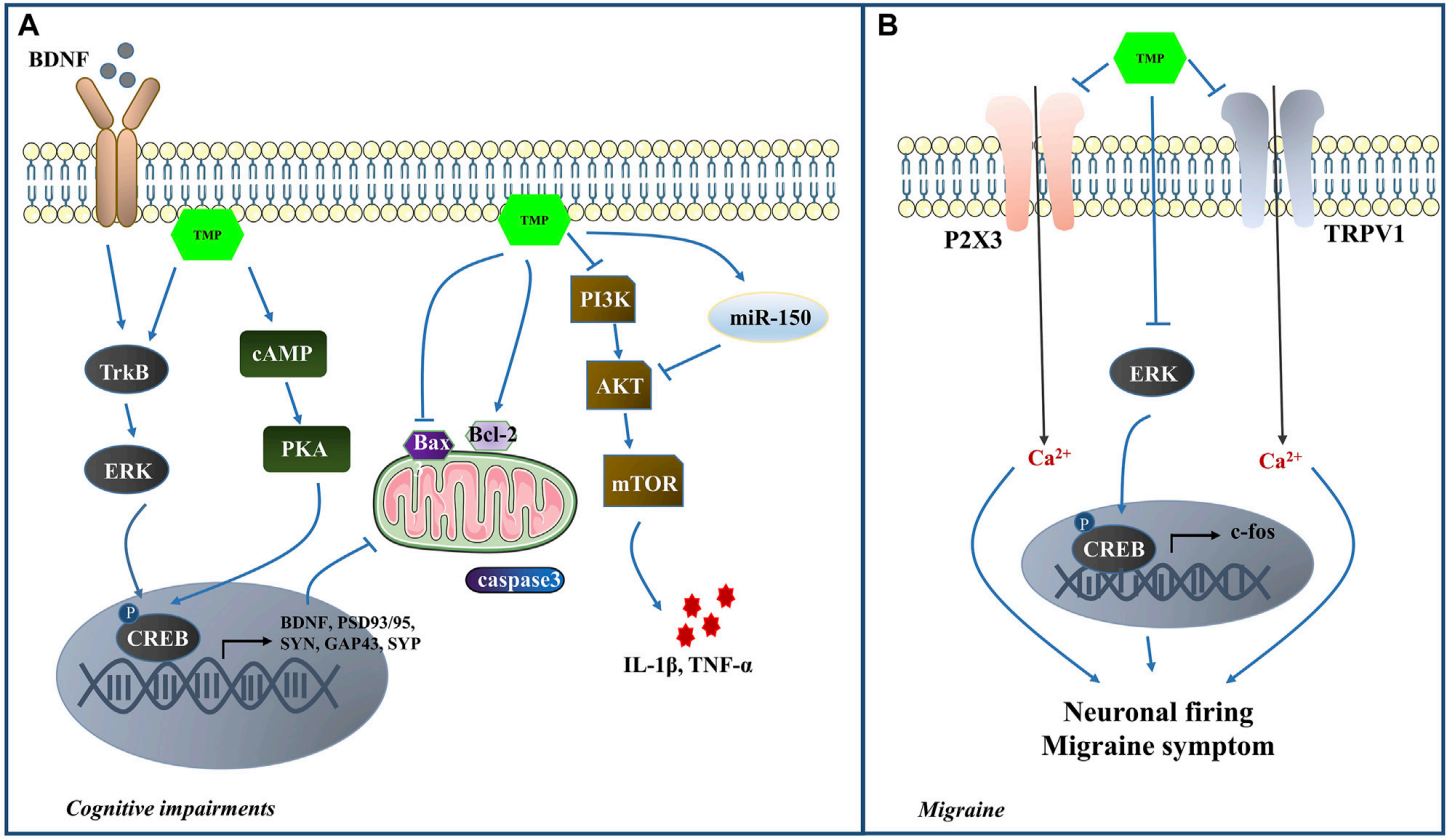
**(A)** The mechanisms of TMP intervention in CI; **(B)** The mechanisms of TMP intervention in MI.

TMP has been effective in alleviating learning and cognitive impairment through various mechanisms. In LPS-induced cognitive impairment model, TMP reduced the inflammatory cells in the brain tissue, suggesting its role in neuroinflammation alleviation and cognitive impairment improvement (Guan et al., 2021). Li et al. (2020b) reported that TMP could activate autophagy by inhibiting the PI3K/Akt/mTOR pathway and further reduce the expression levels of IL-1β and TNF-α, thus mitigating learning/memory function and neurocognitive impairments. MiR-150 is a key anti-inflammatory miRNA that regulates neuroinflammation by targeting Akt3 pathway (Ji et al., 2018; Cai et al., 2019). TMP promoted the modulation of miR-150/Akt pathway *in vivo*, reducing neuroinflammation and improving learning and memory function (Cui et al., 2020). The cAMP/PKA/CREB pathway is critical for memory formation because it promotes synaptic plasticity (Kandel, 2012). Evidence indicates that TMP treatment preserved the expression levels of PSD93 and PSD95 by restoring the normal function of cAMP/PKA/CREB pathway (Wu et al., 2013). Cumulatively these studies indicate that TMP might attenuate CI by suppressing the inflammatory response and apoptosis and promote synaptic plasticity (Figure 4).

### 4.6 Migraine

Migraine (MI), classified clinically as a primary headache, ranks second among disabling medical illnesses worldwide, according to the 2017 Global Burden of Disease Survey (Global, 2019). The mainstay of MI treatment involves ergot alkaloids and triptans. However, they can cause severe vasoconstriction (Raut et al., 2020). Further research into new drugs is warranted for the treatment of MI.

Previous studies have demonstrated that the activation of the trigeminovascular system plays a crucial role in MI (Chen and Wu, 2020). The interaction of Purinergic (P2X3) receptors and transient receptor potential vanilloid subtype 1 (TRPV1) in trigeminal sensory neurons contributes to mechanical hyperalgesia (Saloman et al., 2013). In addition, ERK1/2 has an essential role in the occurrence and development of neuropathic pain (Chen et al., 2016). p-ERK is translocated from the cytoplasm into the nucleus, where it exerts its regulatory effects on the target gene expression by phosphorylation of CREB. Finally, the target gene c-fos participates in the course of MI (Tang et al., 2020). Nitroglycerin (NTG) was used in a study to establish the MI rat model and elucidate TMP’s effects on analgesia. The results indicated that TMP could relieve MI symptoms and reduce the duration and severity of MI headaches. The TMP mechanism may be associated with the inhibition of P2X3, TRPV1, c-fos, ERK and p-ERK expression in the NTG-induced MI rats (Li et al., 2021b). In the nociceptive durovascular trigeminal activation induced rat MI model, TMP could inhibit nociceptive dural-evoked neuronal firing in the trigeminocervical complex (Zhao et al., 2018). Thus it is worth exploring the potential of TMP-mediated MI protection (Figure 4).

### 4.7 Depression

Depression is a mood disorder and serious medical illness and it is a significant contributor to the global burden of diseases affecting millions of people worldwide. As reported by World Health Organization that depression will become the second most devastating disease only to cardiovascular disease by year 2030 (Uphoff et al., 2020).

The pathogenesis of depression has not been fully elucidated to date. However, the immune-inflammatory response may have some involvement in the disease progression (Haapakoski et al., 2016). NLRP3 is known to mediate the activation of microglia and the release of inflammatory factors, playing an essential role in the development of depression (Alcocer-Gómez and Cordero, 2014; Wong et al., 2016; Kaufmann et al., 2017). TLR4 on cell membranes recognizes pathogen-associated molecular patterns (PAMPs) or DAMPs and activates NF-κB, which upregulates the mRNA transcription levels of NLRP3, pro-IL-1β and pro-IL-18, thus mediating the inflammatory responses (He et al., 2016). In a chronic unpredictable mild stress (CUMS) -induced mice model, TMP exerted strong anti-inflammatory effects by inhibiting the TLR4 and NLRP3-associated proteins and suppressing the levels of inflammatory cytokines TNF-α, IL-1β, and IL-6 (Fu et al., 2019). Additionally, TMP also remarkably improved the levels of serotonin (5-HT) and norepinephrine (NE) (Fu et al., 2019). It is well established that the inhibition of the ERK pathway contributes to the onset of depression (Wang and Mao, 2019). The reduction in the BDNF and CREB levels, which are respectively the upstream and downstream molecules involved in the ERK pathway, is crucial in the pathogenesis of depression (Wang and Mao, 2019). *In vivo*, TMP was reported to reverse the decrease of BDNF protein and enhance the phosphorylation levels of ERK1/2 and CREB (Jiang et al., 2015). Although there is a lack of suffcient data to support TMP’s involvement in ameliorating depression, current evidence indicates that TMP usage may be a novel strategy in treating depression.

## 5 Conclusion

**FIGURE 5:**
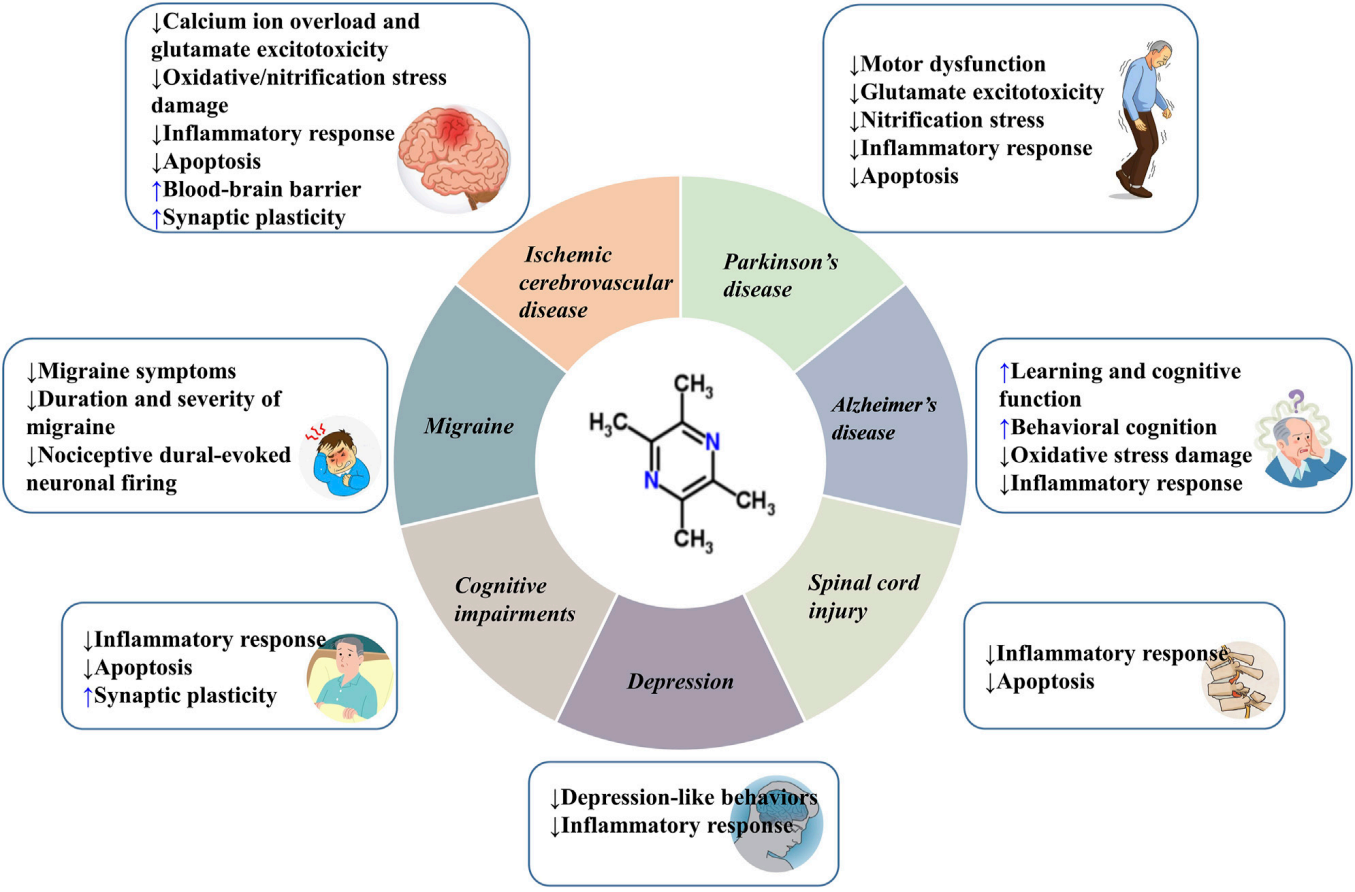
The beneficial efffects of TMP on CNS diseases.

In this paper, we summarize the neuroprotective effects of TMP on various CNS diseases, including ICVD, SCI, PD, AD, CI, MI, and depression. Although the pathological manifestations and mechanisms are different, the protective effects of TMP against these CNS diseases are based on the following aspects: inhibiting the calcium ion overload and glutamate excitotoxicity, oxidative/nitrification stress, inflammatory response, apoptosis, and protecting the integrity of BBB and facilitating synaptic plasticity (Figure 5; Table 1). To improve the pharmacodynamics activities and pharmacokinetic properties, and given the short half-life and low bioavailability of TMP, multiple drug delivery systems and TMP derivatives have been developed to date (Zou et al., 2018). This greatly improves the drugability and clinical application value of TMP. Meta-analysis showed that TMP preparation had high safety, but there are also some adverse reactions, such as dry mouth, vomiting, and dizziness (Yu et al., 2016). Thus, TMP should be used reasonably under the guidance of instructions.

**TABLE 1:** Effects of TMP *in vivo* and *in vitro* studies of CNS diseases.

Type	Inducer	Animal/Cell	Effect	Targets or pathways	References
Ischemic cerebrovascular disease					
*In vitro*	OGD/R	BMECs	↓Calcium ion overload	↓Ca^2+^ overload	Yu et al. (2019a)
*In vivo*	MCAO	Rats	↓Calcium ion overload	↓Ca^2+^ overload	Tang et al. (2012)
*In vivo*	MCAO/R	Rats	↓Glutamate excitotoxicity	↓Glu, Asp	Han et al. (2014)
*In vitro*	OGD/R	BMECs	↓Oxidative stress	↓ROS, MDA; ↑SOD, CAT, GSH-Px	Yu et al. (2019a)
*In vitro*	OGD	BMECs	↓Oxidative stress	↑SOD; ↓LDH	Dave et al. (2016), Yang et al. (2017)
*In vitro*	Glu	PC12 cells	↓ROS, HIF-1α	↓ROS/HIF-1α pathway	Zhang et al. (2018)
*In vitro*	H_2_O_2_	BMSCs	↓Oxidative stress	↓ROS	Yan et al. (2017)
*In vivo*	MCAO	Rats	↓Oxidative stress	↑Trx	Jie et al. (2009)
*In vitro*	OGD	BMECs	↓ROS; ↑eNOS	↓Rho/ROCK pathway	Yang et al. (2017)
*In vitro*	OGD	HAECs	↓ROS; ↑eNOS, NO	↑PI3K/Akt pathway	Ding et al. (2019)
*In vivo*	MCAO	Rats	↓nitrification stress	↓iNOS	Sheu and Hsiao (2006)
*In vivo*	MCAO	Rats	↓oxidative/nitrification stress	↑SOD; ↓MDA, iNOS	Yang et al. (2012)
*In vitro*	TNF-α	HUVECs	↓iNOS, NO	↓IκB kinase (IKK) phosphorylation, IκB degradation, NF-κB nuclear translocation	Zheng et al. (2013)
*In vivo*	MCAO	Rats	↑Neurogenesis	↓nNOS	Xiao et al. (2010)
*In vivo*	CCAO	Rats	↓Inflammatory response	↓ED-1	Liao et al. (2004), Kao et al. (2006)
*In vitro*	LPS/IFN-γ	Glial cells	↓Inflammatory response	↓MPO, PGE2	Liao et al. (2004)
*In vivo*	MCAO	Rats	↓Inflammatory response	↓GFAP, IL-1	Cao et al. (2020)
*In vivo*	MCAO	Mice	↓Inflammatory response	↑MCPIP1; ↓TNF-α, IL-1β, IL-6	Jin et al. (2021)
*In vivo*	MCAO	Rats	↓Inflammatory response	↓IL-1β, caspase-1, NLRP3; ↑PI3K/Akt/Nrf2/HO-1 pathway	Zhu et al. (2022)
*In vivo*	MCAO	Rats	↓Inflammatory response	↓neutrophil migration, HMGB1, TLR4; ↑Nrf2/HO-1 pathway	Chang et al. (2015)
*In vivo*	4-vessel occlusion	Rats	↓Numbers of apoptotic neurons	↑Bcl-2;↓Bax, p53, caspase3	Yu et al. (2017)
*In vitro*	OGD	BMECs	↓Apoptosis	↑Bcl-2; ↓Bax, p53, caspase3	Yu et al. (2019a)
*In vivo*	MCAO and CCAO	Rats	↓Apoptosis	↓DNA fragmentation, ↓caspase-3, Cyt c	Kao et al. (2006), Cheng et al. (2007)
*In vitro*	OGD	Rats cortical neurons	↓Caspase 3	↑TRPC6	Shao et al. (2017)
*In vitro*	Glu	PC12 cells	↑Bcl-2; ↓Bax	↑PKA/CREB pathway	Tong et al. (2022)
*In vitro*	A/R	Rats hippocampal neurons	↓Apoptosis rate of hippocampal neurons	↓JNK/MAPK pathway	Vinken et al. (2006)
*In vitro*	OGD	Rats hippocampal neurons	↓Apoptosis, Cx32	↓ERK1/2, p38-MAPK	Gong et al. (2014)
*In vivo*	MCAO	Rats	↓BBB permeability	↑Occludin, claudin-5	Tan et al. (2015a)
*In vivo*	MCAO	Rats	↑TJ protein	↓JAK/STAT pathway	Gong et al. (2019)
*In vivo*	MCAO	Rats/mice	↓BBB permeability	↓MMP-9	Jin et al. (2021), Tan et al. (2015b)
*In vivo*	MCAO	Rats	↑Synaptic plasticity	↑SYP, GAP-43	Lin et al. (2021)
*In vivo*	MCAO	Rats	↑Synaptic plasticity	↑MAP-2	Lin et al. (2015)
Spinal cord injury					
*In vivo*	Modified Allen’s weight drop apparatus	Rats	↓Inflammatory response	↓MIF	Xiao et al. (2012)
*In vivo*	Modified Allen’s weight drop apparatus	Rats	↓Inflammatory response	↓MIF, NF-кB, IL-18, neutrophil; ↑NF-κB inhibitor, IL-10	Hu et al. (2013)
*In vivo*	Spinal cord compression injury	Mice	↓Inflammatory response	#2E3033; ↓TNF-α, IL-1β, COX-2	Shin et al. (2013)
*In vivo*	Modified Allen’s weight drop apparatus	#2E3033; Rats	#2E3033; ↓apoptosis	↓miR-214-3p→↑Bcl2l2; ↓TUNEL-positive cells, cleaved caspase 3, Bax	Fan and Wu (2017)
*In vivo*	Modified weight-drop device	#2E3033; Rats	#2E3033; ↓apoptosis	↑miR-21→↓FasL, PTEN, PDCD4	Huang et al. (2016)
Parkinson’s disease					
*In vivo*	MPTP	Rats	↓Motor dysfunction	↑TH, DA, DOPAC	Lu et al. (2014)
*In vivo*	MPTP	#2E3033; Rats	#2E3033; ↓apoptosis	↑GSH, Bcl-2; ↓Bax, Cyt c, caspase3	Lu et al. (2014)
*In vivo*	MPTP	Mice	↓Inflammatory response; ↓glutamate excitotoxicity	↓TNF-α, IL-1β, glutamatergic transmission	Zhao et al. (2014)
*In vivo*	Rotenone	#2E3033; Rats	#2E3033; ↓apoptosis; ↓inflammatory response; ↓nitrification stress	↓Bax/Bcl-2, caspase 3, NF-кB, COX2, GFAP, iNOS	Michel et al. (2016)
Alzheimer’s disease					
*In vivo*	APP/PS1	Mice	↑Learning and cognitive function	↓Aβ deposition, tau phosphorylation	Huang et al. (2021), Zhang et al. (2021)
*In vivo*	#212121; Streptozotocin	Rats	↓Aβ deposition, tau phosphorylation	↓GSK-3β	Lu et al. (2017)
*In vitro*	Aβ_25-35_ and IFN-γ	Microglial cells	↓Inflammatory response; ↓oxidative stress	↓TNF-α, IL-1β, MCP-1, NF-κB, ROS	Kim et al. (2014)
*In vivo*	APP/PS1	Mice	↑Behavioral cognition	↑SSTR4	Weng et al. (2021)
*In vivo*	#212121; Scopolamine	Rats	↓Oxidative stress	↑SOD, GSH; ↓MDA	Shi et al. (2012)
Cognitive impairments					
*In vivo*	BCCAO	#2E3033; Rats	#2E3033; ↓Apoptosis	↓MCP-1, Hcy; ↑BDNF	Zhao et al. (2017)
*In vitro*	OGD	PC12 cells	#2E3033; ↓Apoptosis	↓Bax/Bcl-2, caspase 3	Zhao et al. (2017)
*In vivo*	BCCAO and CRS	Rats	↑PSD95, SYN, GAP43, SYP	↑TrkB/ERK/CREB signaling pathway	Tan et al. (2021)
*In vivo*	LPS	Rats	↓Inflammatory response	↓Inflammatory cells	Guan et al. (2021)
*In vivo*	LPS	Rats	↓IL-1β, TNF-α	↓PI3K/AKT/mTOR pathway→↑Autophagy	Li et al. (2020b)
*In vivo*	Isoflurane	Rats	↓IL-1β, IL-6, TNF-α	↓miR-150→↑AKT3	Cui et al. (2020)
*In vivo*	Scopolamine	Rats	↑PSD93, PSD95,	↑cAMP/PKA/CREB pathway	Wu et al. (2013)
Migraine					
*In vivo*	NTG	Rats	↓Migraine symptoms; ↓the duration and severity of migraine headache	↓P2X3, TRPV1, c-fos, ERK, p-ERK	Li et al. (2021b)
*In vivo*	Nociceptive durovascular trigeminal activation	Rats	↓Nociceptive dural-evoked neuronal firing	—	Zhao et al. (2018)
Depression					
*In vivo*	CUMS	Mice	↓TLR4, NLRP3-associated proteins, TNF-α, IL-1β, IL-6	↓TLR4-NF-κB-NLRP3 pathway	Fu et al. (2019)
*In vivo*	CUMS	Mice	↓Depression-like behaviors	↑5-HT, NE	Fu et al. (2019)
*In vivo*	CSDS	Mice	↑BDNF	↑ERK/AKT-CREB pathway	Jiang et al. (2015)

Abbreviations: oxygen-glucose deprivation/reperfusion (OGD/R); brain microvascular endothelium cells (BMECs); middle cerebral artery occlusion (MCAO); rat pheochromocytoma (PC12); hydrogen peroxide (H2O2); bone marrow-derived mesenchymal stem cells (BMSCs); human amniotic epithelial cells (HAECs); human umbilical vein endothelial cells (HUVECs); common carotid arteries occlusion (CCAO); lipopolysaccharide (LPS); interferon -γ (IFN-γ); anoxia/reoxygenation (A/R); 1-methyl-4-phenyl-1,2,3,6-tetrahydropyridine (MPTP); amyloid precursor protein(APP)/presenilin-1(PS1); bilateral common carotid artery occlusion (BCCAO); chronic restraint stress (CRS); nitroglycerin (NTG); chronic unpredictable mild stress (CUMS); chronic social defeat stress (CSDS); decrease (↓); increase (↑).

Currently, the injection of the traditional Chinese patent medicine ligustrazine hydrochloride has been widely used to treat ischemic cerebrovascular disease in China. However, the acceptance and usage of TMP in other countries are still limited. The evidence reviewed in this paper provides possibilities for the widespread clinical application of TMP as a promising therapeutic agent for CNS diseases.

Though TMP has shown promising potential in treating CNS diseases, some lacunae need to be addressed in future studies. First, it should be noted that the evidence mentioned in this paper is mainly obtained from cell and animal experiments, which is not enough to support the efficacy of TMP in clinical practice. Hence, conducting elaborate high-quality, large-scale, multicenter, and randomized controlled clinical trials to explore TMP’s effectiveness and safety is essential. Second, the use of modern detection technologies to explore the pharmacokinetic characteristics of TMP in the CNS is necessary. Also, various drug delivery systems that provide TMP with good solubility, high bioavailability, stable metabolism, and significant biological activity must be assessed. Third, modern molecular biology technologies are developing rapidly, so future research can further study the specific underlying mechanisms of TMP from the perspectives of multiomics and epigenetic modification. In conclusion, more detailed evidence is expected to support the benefical effects of TMP, in order to promote its clinical application worldwide.
